# The new complement inhibitor CRIg/FH ameliorates lupus nephritis in lupus-prone MRL/lpr mice

**DOI:** 10.1186/s12882-019-1599-0

**Published:** 2019-11-21

**Authors:** Yu Shi, Wen Yao, Li Sun, Guomin Li, Haimei Liu, Peipei Ding, Weiguo Hu, Hong Xu

**Affiliations:** 1 Division of Rheumatology, Children’s Hospital of Fudan University, Shanghai, 201102 China; 2 Fudan University Shanghai Cancer Center and Institutes of Biomedical Sciences, Fudan University, Shanghai, 200032 China

**Keywords:** MRL/lpr, Complement C3 inhibitor, Lupus nephropathy

## Abstract

**Backgrounds:**

The aberrant activation of complement system is critically involved in lupus nephropathy. Recent study showed complement C3 inhibitor was effective in the treatment of lupus nephropathy. In this study, we investigate the effect of a novel complement C3 inhibitor, CRIg/FH, in the treatment of lupus nephropathy in MRL/lpr lupus mice.

**Methods:**

We treated MRL/lpr female mice with a dose escalation of CRIg/FH (10, 5 and 2 mg/kg) by intraperitoneal injection twice weekly since 12 weeks age. In addition, MRL/lpr mice treated with intraperitoneal injection of normal saline or oral prednisone, along with C57BL/6 J healthy mice were maintained to serve as controls. We started 8-h urine collection weekly to screen proteinuria by measuring the levels of urine urea/creatinine. Serum samples was collected at week 16 and 20 to measure levels of urea nitrogen, creatinine, and immunological markers (C3, C4, A-ds-DNA) before the mice were sacrificed at 20 weeks age to collect kidneys for histopathological examinations.

**Results:**

Overt skin lesions were observed in MRL/lpr mice treated with normal saline, while skin lesion was not observed in CRIg/FH treated MRL/lpr mice. There was no overt proteinuria observed in MRL/lpr mice treated with CRIg/FH. Serum creatinine and BUN levels in MRL/lpr mice was maintained in highest CRIg/FH dose (10 mg/kg twice a week) to be significantly lower than that in prednisone treated MRL/lpr mice at 20 weeks age. In addition, CRIg/FH treatment in MRL/lpr mice results in a significantly elevated serum C3 and C4 levels when compared to prednisone treatment at both 16 and 20 weeks. Furthermore, our study identified that serum level of A-ds-DNA was also significantly lower in CRIg/FH treatment than that in predisone treated MRL/lpr mice. Renal pathology confirmed that kidneys from CRIg/FH treated MRL/lpr mice suffered less from nephritis and complement disposition.

**Conclusion:**

Our results showed that the complement inhibitor CRIg/FH can protect MRL/lpr mice from lupus nephropathy by preserving renal function and glomerulus complement activation. Our findings support the positive effect of complement inhibitors in the treatment of lupus nephropathy.

Yu Shi and Wen Yao contributed equally to this work.

## Background

Lupus nephropathy (LN) is a common however severe manifestation in systemic lupus erythematosus (SLE) with significant morbidity and mortality [1]. The pathogenesis of lupus LN is initiated by the abnormal activation of complement system triggered by the nephrotic deposition of circulating immune complexes (CIC) formed by autoantibodies in SLE [2]. The activation of complement system in SLE is characterized by the consumption of complement proteins [3–5]. The degree of reduction in serum levels of C1q, C2, C3 and C4, which are components in the classical complement pathway, is associated with the occurrence, development and prognosis of LN [6]. In addition, the activation of the alternative pathway is another important factor that exaggerating complement activation [7]. These evidence indicated that both classical pathway and alternative pathway are involved in the pathogenesis of LN. The aberrantly activated complement leads to complement deposition in the glomeruluss, thereafter the induction of proliferation in glomerular mesangial cells, and finally results in overt nephropathy [8].

The classical treatment of SLE involves the use of nonsteroidal anti-inflammatory drugs, antimalarial drugs, glucocorticoids. However, the broad effect of these systemic immunosuppressants restricts its clinical use owing to difficulties in the balance for attainment of treatment effects over avoidance from adverse reactions. In recent years, complement-targeted therapy (complement inhibitors) showed promising results in the treatment of both SLE and LN [9, 10]. An example of the first generation of complement inhibitors is Eculizumab, a monoclonal antibody that prevents complement C5 from activation through its cleavage into C5a and C5b [11]. In addition, recent studies revealed that inhibition in the early level of complement cascade, complement C3, also has potential therapeutic effects in LN [12]. Nevertheless, concerns regarding C5 or C3 inhibition was often raised due to its effect of systematic inhibition in complements therefore increased risk of both severe infection and opportunistic infection [13].

The CRIg/FH is a novel complement inhibitor and a fusion protein combining the extracellular domain of CRIg and the alternative pathway inhibitory domain of factor H (FH) [14]. The design for CRIg/FH is based on its ability to bind C3 digestion product C3b/iC3b/C3c to avoid unnecessary cells surface deposition, and at the same time, FH domain facilitates factor I-mediated C3b degradation, thereby inhibiting activation and amplification of the alternative pathway [15, 16]. A recent study showed that CRIg/FH can inhibit Thy-1 antibody-mediated complement activation in a rat model of mesangioproliferative glomerulonephropathy (MPGN) therefore protect glomerular mesangial cells from complement-mediated damage and proliferative lesions [14]. These evidence suggested that CRIg/FH could be effective in the treatment of LN in which the activation of complement system is critically involved. Hence in this study we conducted a mice study with a dose escalation treatment of CRIg/FH on the widely used MRL/lpr lupus mice, to investigate the effect of CRIg/FH on lupus nephropathy.

## Materials and methods

### Mice and treatment

Forty female MRL/lpr mice (weighing 37.7 ± 1.6 g at 8 weeks old) from Shanghai Slack Laboratory Animal Co.Ltd. were maintained in the Experimental Animal Science Department of Fudan University under SPF conditions and a 12-h light-dark cycle with free access to standard diet and tap water. At 12 weeks age, the MRL/lpr mice were randomly assigned into treatment groups (*N* = 8 in each group) to receive twice weekly intraperitoneal injection of CRIg/FH [14] with a dose escalation design (10, 5 and 2 mg/kg each injection). The remaining MRL/lpr mice were randomly devided into control groups and received intraperitoneal injection of normal saline (NS) twice a week (group NS, *n* = 8) or daily oral gavage of prednisone (18.2 mg/kg/d, group Pred, n = 8). In addition, eight C57BL/6 J mice from Shanghai Slack Laboratory Animal Co.Ltd. were maintained under same condition and left untreated to serve as normal control.

The mice were monitored for skin lesions, lymphadenopathy, proteinuria, serum creatinine and urea nitrogen, and levels of serum C3, C4 and A-ds-DNA. The study was approved by the institutional review board of Children’s Hospital of Fudan University ([2016]172).

### Urine and serum biochemistry, and serum immunology

During the experiment, 24-h urine samples were collected weekly starting at 12 weeks of age. Proteinuria was detected by measurement of ratio between protein (RenjieBio,Shanghai) and creatinine (Yaoyunbio, Shanghai) in urine samples using ELISA. Blood samples (200 μl) were drawn through tail vein at 16 weeks of age and at 20 weeks of age (time of sacrifice). The serum levels of creatinine, urea nitrogen and the levels of serum C3, C4 and A-ds-DNA were detected by ELISA (Yaoyunbio, Shanghai).

### Histopathological analysis

At age 20 weeks, all mice were sacrificed to collect kidneys after euthanasia by intraperitoneal injection of pentobarbital (200 mg/kg). The kidneys were formalin-fixed and paraffin-embedded and sliced at 4 μm thickness for HE staining. Immunohistochemistry of C3d, membrane attack complex (MAC) and C1q staining were conducted using antibody against C3d (R&D biosystems; AF2655), C5b-9 (Abcam; ab55811), and C1q (Abcam; ab71089). In addition, Fluorescent-dye conjugated antibodies against mouse IgG (Invitrogen; A11029) and mouse IgM mu chain (Abcam; ab150121) were used for immunofluorescence detection of IgG and IgM deposition in the kidney. Renal pathology indicators were scored by two experienced renal pathologists (HL and GL) independently based on the average score according to the activity index of renal tissue in lupus nephropathy [17]. The intensity of immunostaining was reported by GL and blindedly assessed by HL.

### Statistical analysis

The statistical analysis were performed with SPSS version 19.0 software. Continuous variables are presented as mean ± standard deviation. Difference between groups was determined using one-way ANOVA, and a *p* value less than 0.05 was considered to be statistically significant.

## Results

### General conditions

**Fig. 1:**
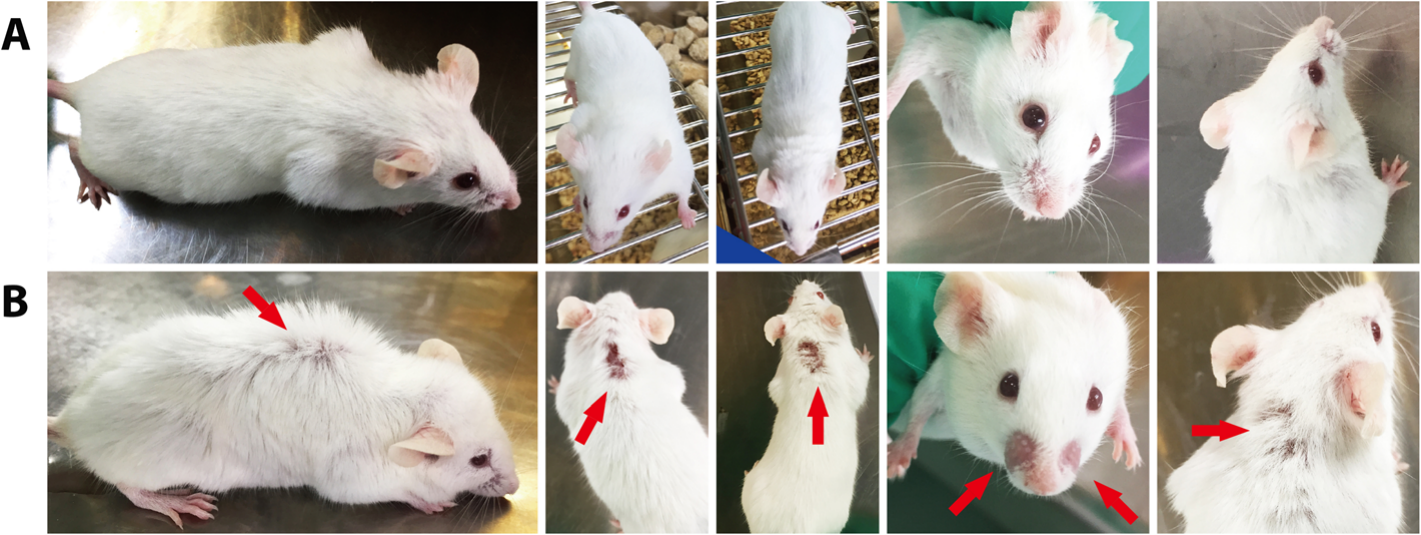
Treatment of MRL/lpr mice with CRIg/FH inhibits the appearance of skin lesions. A 20-week old MRL/lpr mice treated by intraperitoneal injection of 10 mg/kg CRIg/FH twice a week since 12 week age; B 20-week old MRL/lpr mice treated by intraperitoneal injection of normal saline, skin lesions are indicated by red arrows

The normal saline (NS) treated MRL/lpr mice began to develop skin lesion at 16 weeks of age. The skin lesion was overt at 20 weeks of age (Fig. 1). In contrast, MRL/lpr mice treated by 10 mg/kg CRIg/FH twice weekly presented less hair loss.There were two mice died in normal saline treated MRL/lpr mice and one died in oral prednisone treated MRL/lpr mice before 20 weeks age..

### CRIg/FH reduce proteinuria and protect renal function in MRL/lpr mice

**Fig. 2:**
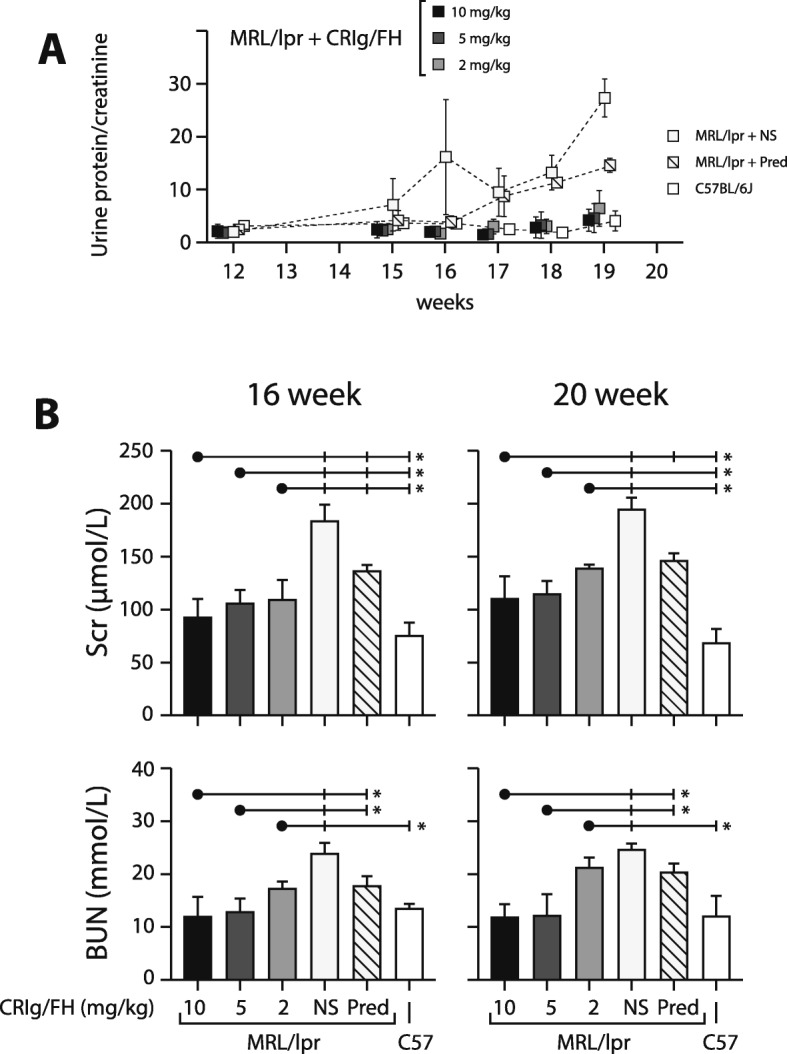
Proteinuria and renal function in MRL/lpr mice. A urine protein/creatinine ratio of studied mice; B levels of serum creatinine and blood urea nitrogen at 16 and 20 weeks age of studied mice. *P < 0.05 compared between CRIg/FH treated mice (black dot) and each control group (vertical bar). NS = normal saline; Pred = prednisone; Scr = serum creatinine; BUN = blood urea nitrogen. The CRIg/FH treatment at indicated dose was administrated twice a week

The level of proteinuria in either NS treated or prednisone treated MRL/lpr mice were significantly elevated after 16 weeks of age (Fig. 2a), whereas protein levels in urine of MRL/lpr mice treated with CRIg/FH were still not significantly elevated by 20 weeks of age. After 18 weeks of age, the level of proteinuria in all dosage groups of CRIg/FH treated MRL/lpr mice were significantly lower than that of the NS or GC treated mice (*P* < 0.05, Fig. 2a).

Treatment with CRIg/FH significantly reduced serum creatinine and urea nitrogen levels in MRL/lpr mice. At 16 weeks of age, serum creatinine levels in all CRIg/FH treated MRL/lpr mice were significantly lower than those in either NS or prednisone treated MRL/lpr mice (*P* < 0.05, Fig. 2b). At 20 weeks, serum creatinine levels in CRIg/FH treatment groups maintained to be significantly lower than that in NS treated MRL/lpr mice (*P* < 0.05). However, comparing to prednisone treated MRL/lpr mice, significant decrease in serum creatinine level was only observed in CRIg/FH treatment with highest dose (10 mg/kg twice weekly, *P* < 0.05, Fig. 2b).

Similarly, serum urea nitrogen levels in MRL/lpr mice treated with CRIg/FH were all significantly lower than those in the NS treated MRL/lpr mice at 16 weeks of age (*P* < 0.05, Fig. 2b). In addition, serum urea nitrogen level in CRIg/FH treatment with higher dosage (10 and 5 mg/kg twice weekly) were significantly lower than that in the prednisone treated MRL/lpr mice (P < 0.05, Fig. 2b). At 20 weeks of age, the effect of CRIg/FH to maintain serum urea nitrogen at low levels in MRL/lpr mice was observed in higher dosage (10 and 5 mg/kg twice weekly) however not in the lowest dose (2 mg/kg) group (Fig. 2b), .

### CRIg/FH blocks complement activation in MRL/lpr mice

**Fig. 3:**
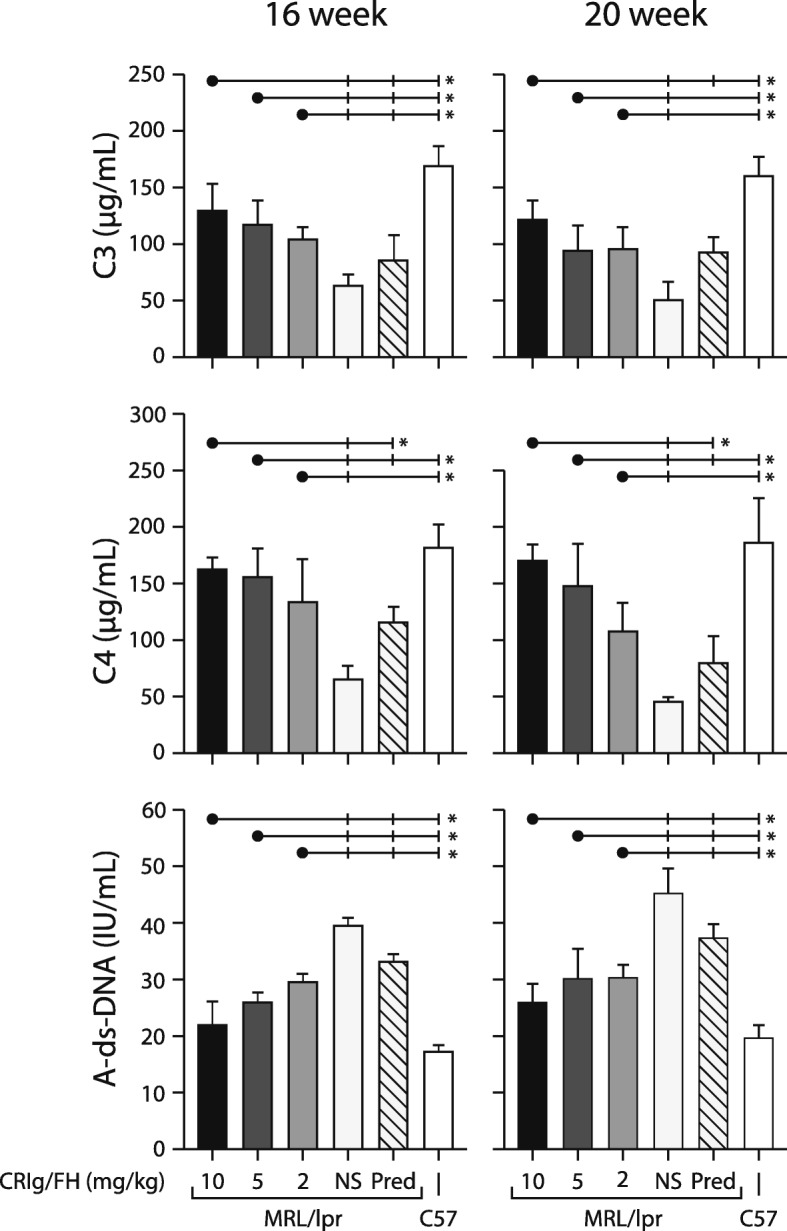
Levels of serum complements (C3, C4) and lupus autoimmune marker (A-ds-DNA) in MRL/lpr mice. *P < 0.05 compared between CRIg/FH treated mice (black dot) and each control group (vertical bar). NS = normal saline; Pred = prednisone; The CRIg/FH treatment at indicated dose was administrated twice a week

At 16 weeks, the levels of serum complement C3 and C4 in MRL/lpr mice treated with CRIg/FH at all dose was significantly higher than that in NS treated MRL/lpr mice (Fig. 3). The difference in C3 was maintained at 20 weeks age mice only between MRL/lpr mice treated with highest dose CRIg/FH (10 mg/kg twice weekly) and NS treated MRL/lpr mice (Fig. 3). Nevertheless, the levels of serum C3 and C4 in CRIg/FH treated mice was significantly lower than that in C57BL healthy mice at both 16 and 20 weeks age (Fig. 3). The serum levels of C4 was significantly lower in higher dose (10 and 5 mg/kg twice weekly) of CRIg/FH treated MRL/lpr mice when compare to either NS or prednisone treated MRL/lpr mice, at both week 16 and 20. The significance of C4 decreasing is not identified in lowest dose of CRIg/FH (2 mg/kg twice weekly) when compared to prednisone treated MRL/lpr mice (Fig. 3).

Similarly, the activation of autoimmunity was significantly lower in MRL/lpr mice treated with CRIg/FH. At 16 weeks, the serum A-ds-DNA level in MRL/lpr mice treated with CRIg/FH at all doses was significantly lower than that in NS treated MRL/lpr mice or GC treated MRL/lpr mice (Fig. 3). The effect continued at 20 weeks measurement (Fig. 3). Nevertheless, the levels of serum A-ds-DNA was still significantly higher in CRIg/FH treated MRL/lpr mice than that in normal control mice at both 16 and 20 weeks (Fig. 3).

### CRIg/FH improves lupus nephropathy in MPL/lpr mice

**Fig. 4:**
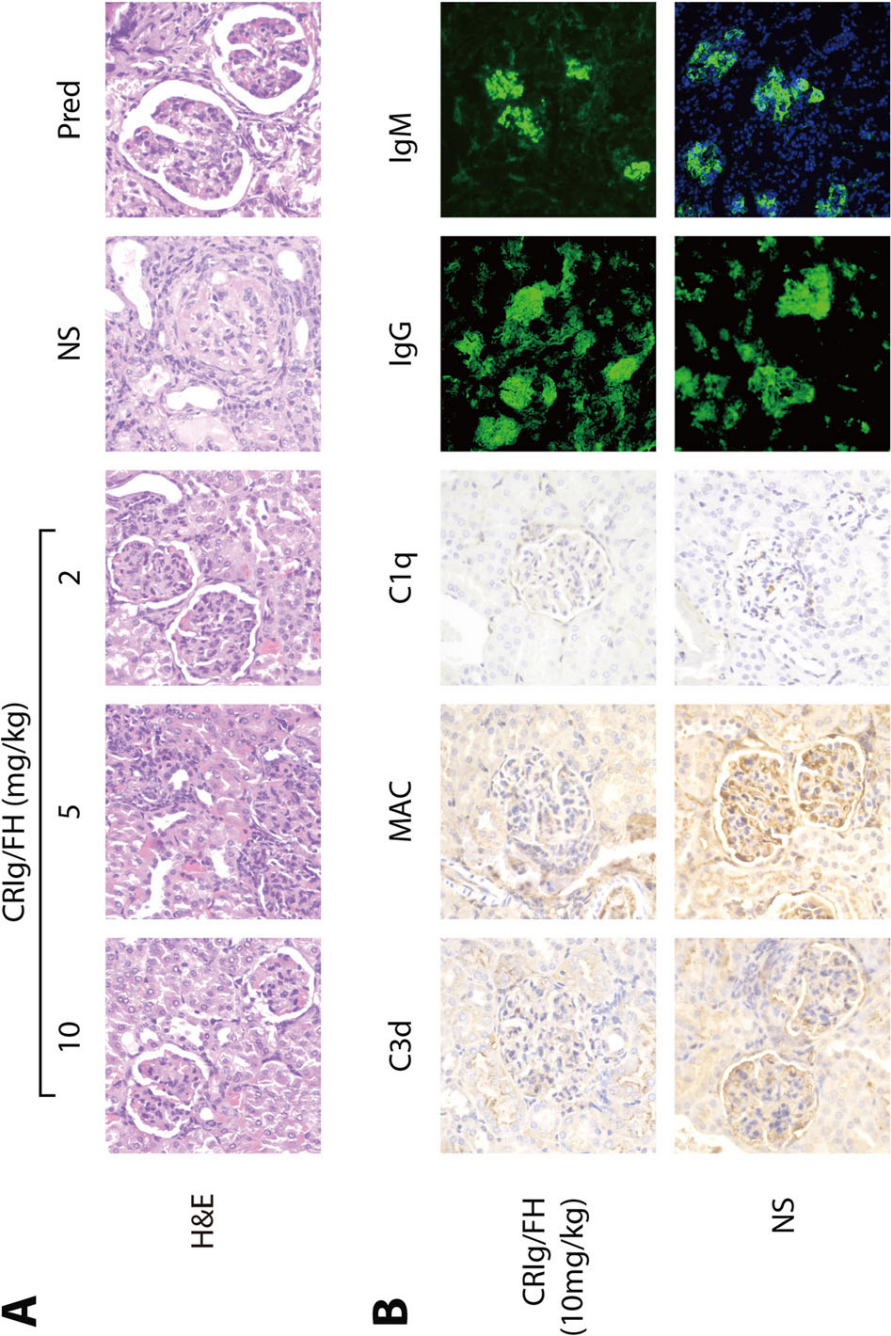
Renal histopathology in MRL/lpr mice. **a** Histopathological features of the studied mouse renal tissues determined by H&E. **b** Immunohistological findings for renal tissues from MRL/lpr mice treated by CRIg/FH at 10 mg/kg twice weekly and normal saline (NS). The NS treated mice showed significant glomerular atrophy, basement membrane thickening and rupture, mesangial area widening, mesangial matrix increases, mesangial cell proliferation and deposition of components of immunopathogenesis; MRL/lpr mice revealed less renal damage and immunological deposition. NS = normal saline, Pred = prednisone, MAC = membrane attack complex. The CRIg/FH treatment at indicated dose was administrated twice a week

The H&E staining of mice kidney at 20 weeks of age showed that MRL/lpr mice had significant nephropathy in those treated by NS than CRIg/FH (Fig. 4a). In MRL/lpr mice treated with highest dose CRIg/FH (10 mg/kg twice weekly), there were 7 mice categorized as LN II, 1 mice LN III, whereas all mice treated with NS were scored as LN IV-G. The activity index in MRL/lpr mice treated with CRIg/FH were significantly lower than that in NS treated lupus mice (Table 1). The activity index of MRL/lpr mice treated with highest dose of CRIg/FH was significantly lower than that of prednisone treated MRL/lpr mice (*P* < 0.05). Nevertheless, the nephropathy index of activity was not statistically different between lowest dose of CRIg/FH treatment (2 mg/kg twice weekly) and prednisone treatment. The chronic index of MRL/lpr mice treated with higher dosage of CRIg/FH (10 and 5 mg/kg twice weekly) was the same as that in prednisone treated MRL/lpr mice.

**Table 1:** Renal pathology indicator scores in MRL/lpr mice

	CRIg/FH*				
	10 mg/kg	5 mg/kg	2 mg/kg	prednisone	NS
Activity index	4.3 ± 2.2	7.0 ± 1.2	9.1 ± 1.4	7.8 ± 0.64	14.3 ± 3.5
Chronicity index	0	0	0.13 ± 0.35	0	3.8 ± 3.44

Data are presented in mean ± standard deviation. *CRIg/FH was administrated intraperitoneally with indicated dosage twice a week, NS = normal saline

**Table 2:** Intensity of immunological complements in mice glomerulus

	C3d		MAC		C1q		IgM		IgG	
	CRIg/FH	NS	CRIg/FH	NS	CRIg/FH	NS	CRIg/FH	NS	CRIg/FH	NS
*Intensity of staining*										
–									1	
±	1				4				4	
+	7	7	6	3	4	3		3	3	4
++		1	2	4		5	6	5		4
+++				1			2			

Data are presented as number of mice scored by intensity of immunological staining, in comparison between CRIg/FH (10 mg/kg twice weekly) and normal saline (NS) treated MRL/lpr mice. Kidneys from the two MRL/lpr mice treated by NS who died earlier were also examined

The deposition of complement MAC, C1q, and C3d in all CRIg/FH treatment groups were significantly reduced compared with NS treated mice. Nevertheless, the deposition of immunoglobulin IgM and IgG were not significantly improved in CRIg/FH treatment groups. (Fig. 4b, Table 2).

## Discussion

In this study, we explored the effect of a C3 complement inhibitor, CRIg/FH, on lupus nephropathy using the widely used MRL/lpr lupus mice. Our results showed that CRIg/FH was able to protect in MRL/lpr mice from nephropathy as presenting lower level of proteinuria, improved renal function and serum immunological markers and minor nephritis.

The complement system actively participates in the pathogenesis of lupus nephropathy. The lupus nephropathy is initiated by the nephrotic deposition of abnormal circulating immune complexes (CIC) formed by autoantibodies against free nucleosomes [18]. After CIC inducing complement cascade reaction through classical pathway, the proliferation and activation of glomerular mesangial cells was triggered to form glomerular diseases [19]. The initial activation and further exaggerated complement activation by alternative pathway together contributed to the pathogenesis of overt nephritis in lupus [20]. It was found that monoclonal antibody against C5 effectively improved lupus nephropathy in both lupus mice [21] and patients with lupus nephropathy presenting low levels of serum complement [22]. The C5 blockers which acts effectively against the formation of MAC therefore is currently available and widely used in practice to treat autoimmune diseases [23]. Nevertheless, the complement inhibiting effect of C5 blockers is often extensive and systematic, resulting in elevated risk of immunosuppression and opportunistic infections [24]. Although recently study showed that C3 blocker effectively improved proteinuria and preserved renal function in lupus mice [12], despite its targeting earlier phases in the complement activation, potentency of C3 blocker to broadly suppress complement activation still raised similar concerns [12]. The CRIg/FH is a fusion protein combining the extracellular domain of CRIg (C3b/iC3b/C3c binding domain) and the alternative pathway inhibitory domain of factor H (SCR1–5) [14]. The combination of the two domains was shown to not only bind to the C3 digestion product C3b/iC3b/C3c and subsequent reduce activation of classic pathway, but also inhibit the activation of alternative pathway [15, 16]. The effect of CRIg/FH against complement overactivation induced nephritis was first recognized in rat model of mesangioproliferative glomerulonephropathy (MPGN) [14]. The ability of CRIg/FH to inhibit classical pathway and remove C3b/iC3b deposition on cell surface and protecting glomerular mesangial cells raised the possibility of its treatment effect in other complement activation related nephritis [14]. Additional evidence showed that the binding between CRIg/FH and C3b subunit on the surface of macrophages was able to place FH domains to participant and inhibit alternative pathway [16, 25, 26]. In this study, we found that the administration of complement inhibitor CRIg/FH can reduce the kidney injury in MRL/lpr mice in a dose related manner. Through serum complement profiling, we showed that CRIg/FH prevents the consumption of C3 and C4, suggesting that CRIg/FH act through the inhibition of complement cascade.

The effect of CRIg/FH on lupus nerphritis was confirm by renal pathology and immunofluorescence of the lupus mice. CRIg/FH was able to maintain normal glomerulus morphology and inhibiting levels of local C3 and C4 however not CIC. Interestingly, our results showed that the A-ds-DNA titer decreased in lupus mice treated with CRIg/FH. Although the complement inhibitor does not have directly effect on adaptive immune system, there are preliminary data showing that complement inhibition contributed to the relief of disease activity of lupus in both human subjects and MRL/lpr mice model [27, 28]. Despite its detailed mechanism is currently unexploited, it was suggested that the clinical value of the drug in treating SLE can act beyond the complement system.

## Conclusions

Our study showed that complement inhibitor CRIg/FH can effectively treat lupus nephropathy in the classical MRL/lpr model. The protective effects of lupus nephropathy are manifested in many aspects, including reducing proteinuria, serum creatinine and urea nitrogen levels, and reducing tubular inflammation. In addition to directly blocking the complement pathway of complement, whether CRIg/FH can improve the inflammation of kidney and alleviate the proliferation of mesangial cells through other pathways needs to be further clarified. Further experiments are needed to confirm the relationship between complement and the acquired immune system in SLE.

## Data Availability

The data that support the findings of this study are available from the corresponding author, WH and HX, upon request.
